# Impact of marginalization on characteristics and healthcare utilization among people with substance use disorder in Ontario, Canada, before and during the COVID-19 pandemic: A cross-sectional study

**DOI:** 10.1371/journal.pone.0312270

**Published:** 2024-10-25

**Authors:** Cherry Chu, Bilal Khan, Deva Thiruchelvam, Janette Brual, Ibukun-Oluwa Omolade Abejirinde, Altea Kthupi, Mina Tadrous

**Affiliations:** 1 Women’s College Hospital Institute for Health System Solutions and Virtual Care, Toronto, Ontario, Canada; 2 Institute for Clinical Evaluative Sciences, Toronto, Ontario, Canada; 3 Dalla Lana School of Public Health, University of Toronto, Toronto, Ontario, Canada; 4 Leslie Dan Faculty of Pharmacy, University of Toronto, Toronto, Ontario, Canada; Ulster University - Coleraine Campus: Ulster University, UNITED KINGDOM OF GREAT BRITAIN AND NORTHERN IRELAND

## Abstract

**Objective:**

To describe and compare the characteristics of people with SUD and their use of healthcare services in two ways: 1) across varying levels of marginalization and 2) before and during the pandemic.

**Methods:**

We conducted a population-based cross-sectional study using administrative data from Ontario, Canada. We included individuals age 16+ with a recorded diagnosis of SUD between June 2018–2019 (pre-pandemic) and June 2021–2022 (during-pandemic). Baseline sociodemographic and clinical characteristics and use of healthcare services were enumerated across the five quintiles of the Ontario Marginalization Index.

**Results:**

259,497 pre-pandemic and 276,459 during-pandemic people with SUD were identified. Over 40% belonged to the two highest marginalization quintiles (Q4/Q5). Most had an outpatient visit with similar percentages across quintiles, however the number of visits increased with increasing marginalization (pre-pandemic: mean 8.5 visits in Q1 vs 13.0 visits in Q5; during-pandemic: mean 9.5 in Q1 vs 13.4 in Q5). There was no consistent pattern in percent of people who sought alcohol-related outpatient care, however more marginalized people sought drug-related outpatient care (pre-pandemic: 19.1% in Q1 vs 31.7% in Q5; during-pandemic: 18.7% in Q1 vs 32.5% in Q5). Almost half of people with SUD had an emergency department (ED) visit, of which more belonged to higher marginalization quintiles (pre-pandemic: 43.5% in Q1 vs 49.8% in Q5; during-pandemic: 41.4% in Q1 vs 49.3% in Q5).

**Conclusions:**

SUD prevalence and most health service utilization remained similar from pre- to during-pandemic. Increasing marginalization was associated with increased use of healthcare among people with SUD. Future research should aim to further explore the complex relationship between marginalization and substance use.

## Introduction

Substance use disorder (SUD), defined as a compulsive and continuous use of drugs despite negative impacts to the individual’s life and the lives of others [1], is a major public health concern. It is estimated that 21% of the Canadian population uses substances problematically [2], and potentially more have experienced the harms of substance use even without having SUD. Commonly misused substances are alcohol, cannabis, opioids, cocaine, and methamphetamine, along with prescription medications including prescription opioids, sedatives (i.e., benzodiazepines), and prescription stimulants [3]. Alcohol is the most used substance among Canadians, likely owing to its legalized and socially acceptable status, with over 15% of those who drink engaging in risky drinking behaviour [4]. Alcohol-induced deaths have increased significantly over the past years, with 3,875 deaths recorded in 2021, affecting primarily younger populations [5]. Opioid addiction is also prevalent, with an estimated 14% of Canadians using opioids for medical or non-medical purposes [6]. The drug overdose epidemic, or the opioid crisis, has led to over 34,400 opioid toxicity deaths in Canada during the past 6 years [7].

SUD is often comorbid with mental illness—a meta-analysis found that depression and anxiety was bidirectionally associated with use of alcohol, cannabis, and tobacco [8]. People with substance use and mental health conditions are high users of healthcare services and are more likely than non-affected people to have visits not related to substance use or mental health, contributing to high costs of care for this population [9].

The COVID-19 pandemic further exacerbated problematic substance use and mental health due to stressors such as financial situations, employment precarity, social isolation, as well as diminished access to healthcare services [10, 11]. Marginalized and low-income communities have been particularly impacted by the pandemic, with national surveys reporting 30–50% of low-income or unemployed respondents having problematic substance use [10].

There is abundant literature on characteristics and healthcare utilization among people with SUD [12, 13]. However there has been limited attention on the complex, potential dose-response relationship between marginalization and health outcomes in the SUD population, particularly as it relates to the shifts brought upon by the COVID-19 pandemic. Understanding how marginalization affects the pattern and extent that people with SUD interact with the healthcare system during the pandemic is of importance to decision makers to guide policies and interventions targeted towards vulnerable communities in the post-pandemic recovery period. Therefore, the objective of this study was to compare the sociodemographic and clinical characteristics of people with SUD across varying levels of marginalization in Ontario, Canada’s largest province, as well as their use of healthcare services, before and during the COVID-19 pandemic.

## Methods

We conducted a population-based cross-sectional study describing baseline characteristics and healthcare utilization outcomes among people with substance use disorder in Ontario during a pre-pandemic period and a during-pandemic period. The pre-pandemic period covered June 1, 2018 to June 1, 2019, where June 1, 2018 was defined as the index date (date of cohort entry) for each individual. We compared this cohort to the characteristics and outcomes during a more recent period (June 1, 2021 to June 1, 2022, with index date of June 1, 2021), which reflects a relatively stable time during the pandemic as the healthcare system would have reached equilibrium in terms of the changes brought on by the pandemic.

### Data sources

We used the following health administrative databases from ICES (formerly known as the Institute for Clinical Evaluative Sciences) (see S1 Table): 1) Discharge Abstract Database (DAD), which captures information on all hospital discharges; 2) National Ambulatory Care Reporting System (NACRS), which contains data on all hospital and community-based ambulatory care (including emergency department visits); 3) Ontario Health Insurance Plan (OHIP), which captures data on all physician claims for services provided to patients with healthcare coverage; 4) Ontario Mental Health Reporting System (OMHRS), which captures data on all adults receiving mental health services in the province; 5) Ontario Drug Benefit (ODB) and Narcotics Monitoring System (NMS), which contains records of prescription claims; and 6) Registered Persons Database (RPDB), which captures patient demographic information. Other databases used in this study included Same Day Surgery (SDS), Postal Code Conversion File (PCCF), Ontario Marginalization Index (ONMARG) and various ICES-validated chronic disease registries. All databases were linked using unique encoded identifiers and analyzed at ICES. ICES is an independent, non-profit research institute whose legal status under Ontario’s health information privacy law allows it to collect and analyze health care and demographic data, without requiring consent, for health system evaluation and improvement. The use of the data for this project is authorized under section 45 of Ontario’s Personal Health Information Protection Act (PHIPA) and did not require review by a Research Ethics Board. We obtained an exemption letter from the Women’s College Hospital Research Ethics Board under REB # 2022-0099-A.

### Study design and population

We included individuals aged 16 and above with a recorded diagnosis for substance use disorder [14, 15] within the 5 years before (and including) the index date during the periods June 2018 to June 2019 and June 2021 to June 2022. SUD was defined as meeting any of the following criteria (see S2 Table): a) any emergency department visit or inpatient hospitalization with SUD diagnosis; b) at least 3 claims of an SUD-related outpatient visit; c) any prescription claim for opioid agonist treatment (methadone or buprenorphine) [16].

Eligibility criteria included being alive during the study window, having valid Ontario Health Insurance Plan (OHIP) coverage, and being an Ontario resident.

### Analysis

Baseline characteristics were identified on the index date. The following characteristics were assessed: age, sex, region of residence, rurality, neighbourhood income quintile (higher quintile represents higher income), degree of marginalization for material resources, households and dwellings, racialized and newcomer populations, and age and labour force (measured via the Ontario Marginalization Index with higher quintile representing higher marginalization), history of mental health-related visits in the past 2 years, general physical comorbidity (measured via the Charlson Comorbidity Index with higher value representing more comorbidities), and urine drug screening in the past year.

Healthcare utilization outcomes were captured over the one-year study window (June 1, 2018 to June 1, 2019 for Cohort 1 and June 1, 2021 to June 1, 2022 for Cohort 2). The following outcomes were assessed: emergency department visits, hospitalizations, outpatient visits, prescription medications, and urine drug screening tests. For emergency department visits and hospitalizations, we identified any-cause visits/admissions and those related to mental health, substance use, alcohol use, opioid use, other drug use (e.g., cannabis, sedative/hypnotics, cocaine, other stimulants, hallucinogens, volatile solvents), and other substance use (e.g., nicotine, tobacco, other non-psychoactive substances such as antacids, vitamins). Note that we grouped all remaining drugs or substances into “other” due to low counts. For outpatient visits, we identified any-cause visits and those related to mental health, alcohol dependence, and drug dependence. For prescription medications, we captured all drugs used, drugs related to mental health and addiction, benzodiazepines, opioid agonist treatment (OAT), opioids (non-OAT), and stimulants.

Binary variables were reported as counts and proportions, while continuous variables were reported as means with standard deviations. Because many healthcare utilization outcomes were rare occurrences, we reported most as binary variables (had at least one claim during the study period), with the more common outcomes reported as both proportion of individuals with at least one claim and average number of claims per person.

We report overall population findings with further stratifications based on individuals’ summary quintile across the four domains of marginalization, which was calculated as follows: (households and dwellings quintile + material resources quintile + age and labour force quintile + racialized and newcomer populations quintile) / 4 [17]. We did not conduct any statistical comparisons as we used the entire population of interest and not a sample, and therefore any differences we see in the population are real differences and inferences are made at the population-level. All analyses were conducted using SAS enterprise guide (version 7.1; SAS Institute Inc., Cary, NC).

## Results

In Ontario, 259,497 people with SUD were identified between June 2018 to June 2019 (Cohort 1), and 276,459 were identified between June 2021 to June 2022 (Cohort 2), representing approximately 1.8% of the Ontario population (N = 14,544,701 on July 1, 2019 and N = 15,109,416 on July 1, 2022). Pre-pandemic, 246 (0.9%) of people with SUD were from the lowest marginalization quintile (Q1) and 14,278 (5.5%) were from the highest quintile (Q5), while most people belonged to the middle quintiles (Q2, Q3, Q4). Similarly, during the pandemic, 278 (0.1%) of people were from the lowest quintile and 14,729 (5.3%) were from the highest quintile, with majority of people belonging to the middle quintiles.

### Baseline characteristics

Baseline characteristics in Cohort 1 (pre-pandemic) are reported in Table 1. Mean age generally increased marginally from lower to higher marginalization levels (mean(SD) 39.8(16.3) years in Q1 vs 43.7(16.0) years in Q5). There were almost twice as many males than females with SUD (62.6% vs 37.4%) with 59.3% male in Q1 vs 62.8% in Q5. Q1 had the highest proportion of people with SUD who are rural residents (23.2% vs 76.8% urban) compared to the other quintiles, with the proportion of urban residents increasing as marginalization increases (99.4% urban residents in Q5). A majority of people with SUD had at least one mental health related visit in the past two years (81.6% of total cohort). The proportion of individuals with anxiety (39.9% of total cohort) and mood (20.4% of total) disorders were similar across marginalization quintiles. A small proportion of people with SUD had a psychotic disorder and this percentage appeared to increase slightly from lower to higher marginalization levels (4.5% in Q1 vs 9.7% in Q5). Most people did not have physical comorbidities (90.2% of total cohort), however there were more people with at least one comorbidity in Q5 compared to Q1 (13.9% vs 9.0%).

**Table 1:** Cohort 1 (pre-pandemic): Baseline characteristics stratified by overall marginalization quintile.

Variable	TOTAL	Q1	Q2	Q3	Q4	Q5
	N = 259,497^a^^,^^b^^,^^c^	N = 246	N = 39,070	N = 97,374	N = 98,957	N = 14,278
**Age, mean(SD)**	40.2 (15.7)	39.8 (16.3)	38.4 (16.1)	40.0 (15.8)	41.1 (15.5)	43.7 (16.0)
**Sex, n(%)**						
Male	162,335 (62.6%)	146 (59.3%)	24,653 (63.1%)	61,431 (63.1%)	61,573 (62.2%)	8,965 (62.8%)
Female	97,162 (37.4%)	100 (40.7%)	14,417 (36.9%)	35,943 (36.9%)	37,384 (37.8%)	5,313 (37.2%)
**Rurality, n(%)**						
Urban	225,960 (87.1%)	189 (76.8%)	33,719 (86.3%)	84,142 (86.4%)	92,402 (93.4%)	14,197 (99.4%)
Rural	31,516 (12.1%)	57 (23.2%)	5,351 (13.7%)	13,232 (13.6%)	6,555 (6.6%)	81 (0.6%)
**Neighbourhood income quintile, n(%)**						
1	85,133 (32.8%)	0 (0.0%)	55 (0.1%)	7,402 (7.6%)	58,580 (59.2%)	13,196 (92.4%)
2	55,369 (21.3%)	0 (0.0%)	810 (2.1%)	23,671 (24.3%)	29,420 (29.7%)	1,039 (7.3%)
3	44,522 (17.2%)	8 (3.3%)	4,922 (12.6%)	30,753 (31.6%)	8,555 (8.6%)	43 (0.3%)
4	37,175 (14.3%)	64 (26.0%)	13,634 (34.9%)	21,246 (21.8%)	1,766 (1.8%)	0 (0.0%)
5	35,093 (13.5%)	174 (70.7%)	19,649 (50.3%)	14,302 (14.7%)	636 (0.6%)	0 (0.0%)
**Region of residence, n(%)**						
Central	59,626 (23.0%)	52 (21.1%)	11,670 (29.9%)	24,273 (24.9%)	20,585 (20.8%)	2,518 (17.6%)
East	59,358 (22.9%)	93 (37.8%)	9,988 (25.6%)	21,333 (21.9%)	23,635 (23.9%)	3,216 (22.5%)
North	29,973 (11.6%)	26 (10.6%)	2,446 (6.3%)	10,507 (10.8%)	10,403 (10.5%)	1,010 (7.1%)
Toronto	29,070 (11.2%)	0 (0.0%)	1,713 (4.4%)	13,188 (13.5%)	11,619 (11.7%)	2,112 (14.8%)
West	81,470 (31.4%)	75 (30.5%)	13,253 (33.9%)	28,073 (28.8%)	32,715 (33.1%)	5,422 (38.0%)
**Anxiety disorder, n(%)**	103,535 (39.9%)	96 (39.0%)	16,050 (41.1%)	38,887 (39.9%)	40,063 (40.5%)	5,920 (41.5%)
**Mood disorder, n(%)**	52,902 (20.4%)	54 (22.0%)	7,687 (19.7%)	19,551 (20.1%)	20,966 (21.2%)	3,404 (23.8%)
**Psychotic disorder, n(%)**	17,575 (6.8%)	11 (4.5%)	1,988 (5.1%)	5,931 (6.1%)	7,612 (7.7%)	1,390 (9.7%)
**Any mental health visits in past 2 years, n(%)**	211,646 (81.6%)	205 (83.3%)	31,097 (79.6%)	78,455 (80.6%)	82,089 (83.0%)	12,000 (84.0%)
**Charlson comorbidity index, n(%)**						
0	234,086 (90.2%)	224 (91.0%)	36,231 (92.7%)	88,614 (91.0%)	88,155 (89.1%)	12,301 (86.1%)
1–2	19,078 (7.4%)	13 (5.3%)	2,173 (5.6%)	6,557 (6.7%)	8,102 (8.2%)	1,450 (10.2%)
2+	6,333 (2.4%)	9 (3.7%)	666 (1.7%)	2,203 (2.3%)	2,700 (2.7%)	527 (3.7%)
**Any urine drug screening test in past year, n(%)**	67,234 (25.9%)	52 (21.1%)	7,987 (20.4%)	23,522 (24.2%)	28,940 (29.2%)	4,273 (29.9%)

^a^ Includes N = 9,572 with missing marginalization score (not reported)

^b^ Rurality missing for n = 2,021 (0.8%)

^c^ Neighbourhood income quintile missing for n = 2,205 (0.8%)

Cohort 2 (during pandemic) baseline characteristics are reported in Table 2. Mean age was similar across quintiles with Q5 having the highest mean(SD) age of 44.0(15.8) years. Similar to cohort 1, there were more males than females (62.5% vs 37.5%) with 57.9% male in Q1 vs 62.2% male in Q5. Q1 also had the highest rural to urban ratio (22.7% rural vs 77.3% urban residents) compared to other quintiles, with Q4 and Q5 having the highest urban population (92.8% and 99.4% respectively). Similar proportions of people had anxiety and mood disorders across quintiles (39.5% and 19.5% of the total cohort respectively). Q2 had the lowest proportion of people with psychotic disorder (5.8%) while Q5 had the highest (10.9%). Over three-quarters of the population had at least one mental health visit in the past two years, with the highest percentage found in Q5 (83.1%). Most people did not have physical comorbidities (90.0% of total cohort) however the percentage of people with at least one comorbidity grew marginally with increasing marginalization level (10.1% in Q1 vs 13.7% in Q5).

**Table 2:** Cohort 2 (during pandemic): Baseline characteristics stratified by overall marginalization quintile.

Variable	TOTAL	Q1	Q2	Q3	Q4	Q5
	N = 276,459^a^^,^^b^^,^^c^	N = 278	N = 42,543	N = 104,703	N = 103,137	N = 14,729
**Age, mean(SD)**	41.1 (15.5)	42.3 (16.9)	39.6 (16.0)	41.0 (15.6)	41.9 (15.3)	44.0 (15.8)
**Sex, n(%)**						
Male	172,832 (62.5%)	161 (57.9%)	27,250 (64.1%)	66,053 (63.1%)	63,633 (61.7%)	9,158 (62.2%)
Female	103,627 (37.5%)	117 (42.1%)	15,293 (35.9%)	38,650 (36.9%)	39,504 (38.3%)	5,571 (37.8%)
**Rurality, n(%)**						
Urban	238,082 (86.1%)	215 (77.3%)	36,573 (86.0%)	89,502 (85.5%)	95,721 (92.8%)	14,638 (99.4%)
Rural	36,037 (13.0%)	63 (22.7%)	5,970 (14.0%)	15,201 (14.5%)	7,416 (7.2%)	91 (0.6%)
**Neighbourhood income quintile, n(%)**						
1	89,042 (32.2%)	0 (0.0%)	52 (0.1%)	7,912 (7.6%)	60,535 (58.7%)	13,652 (92.7%)
2	59,121 (21.4%)	0 (0.0%)	904 (2.1%)	25,689 (24.5%)	31,062 (30.1%)	1,030 (7.0%)
3	47,557 (17.2%)	6 (2.2%)	5,427 (12.8%)	32,814 (31.3%)	8,983 (8.7%)	47 (0.3%)
4	40,613 (14.7%)	77 (27.7%)	14,992 (35.2%)	23,116 (22.1%)	1,868 (1.8%)	0 (0.0%)
5	37,597 (13.6%)	195 (70.1%)	21,168 (49.8%)	15,172 (14.5%)	689 (0.7%)	0 (0.0%)
**Region of residence, n(%)**						
Central	62,434 (22.6%)	56 (20.1%)	12,607 (29.6%)	25,334 (24.2%)	21,200 (20.6%)	2,571 (17.5%)
East	63,496 (23.0%)	104 (37.4%)	10,819 (25.4%)	23,299 (22.3%)	24,815 (24.1%)	3,357 (22.8%)
North	33,392 (12.1%)	25 (9.0%)	2,748 (6.5%)	11,538 (11.0%)	11,464 (11.1%)	1,018 (6.9%)
Toronto	29,275 (10.6%)	0 (0.0%)	1,707 (4.0%)	13,477 (12.9%)	11,462 (11.1%)	2,046 (13.9%)
West	87,862 (31.8%)	93 (33.5%)	14,662 (34.5%)	31,055 (29.7%)	34,196 (33.2%)	5,737 (39.0%)
**Anxiety disorder, n(%)**	109,295 (39.5%)	114 (41.0%)	17,498 (41.1%)	42,238 (40.3%)	40,668 (39.4%)	5,918 (40.2%)
**Mood disorder, n(%)**	53,878 (19.5%)	56 (20.1%)	8,019 (18.8%)	20,451 (19.5%)	20,717 (20.1%)	3,288 (22.3%)
**Psychotic disorder, n(%)**	21,329 (7.7%)	23 (8.3%)	2,478 (5.8%)	7,349 (7.0%)	8,981 (8.7%)	1,599 (10.9%)
**Any mental health visits in past 2 years, n(%)**	221,053 (80.0%)	218 (78.4%)	33,032 (77.6%)	83,020 (79.3%)	83,739 (81.2%)	12,241 (83.1%)
**Charlson comorbidity index, n(%)**						
0	248,815 (90.0%)	250 (89.9%)	39,232 (92.2%)	94,940 (90.7%)	91,789 (89.0%)	12,713 (86.3%)
1–2	20,752 (7.5%)	*23–27	2,541 (6.0%)	7,293 (7.0%)	8,505 (8.2%)	1,454 (9.9%)
2+	6,892 (2.5%)	*1–5	770 (1.8%)	2,470 (2.4%)	2,843 (2.8%)	562 (3.8%)
**Any urine drug screening test in past year, n(%)**	64,057 (23.2%)	41 (14.7%)	7,748 (18.2%)	22,853 (21.8%)	26,770 (26.0%)	4,014 (27.3%)

^a^ Includes N = 11,069 with missing marginalization score (not reported)

^b^ Rurality missing for n = 2,340 (0.8%)

^c^ Neighbourhood income quintile missing for n = 2,529 (0.9%)

*Small cells (n<5) are suppressed to prevent re-identification of individuals

S3 Table shows the percentage of people with SUD as a fraction of the population in each region (whereas the tables above show the percentage of people with SUD from each region as a fraction of all people with SUD). We note that in both Cohorts 1 and 2, the top region where people with SUD were from was the West (31.4% and 31.8%, respectively), however the region with the largest proportion of people with SUD was the North (3.7% and 4.2%, respectively).

### Healthcare utilization outcomes

Healthcare utilization for Cohort 1 (pre-pandemic) is reported in Table 3. Over half of people with SUD had at least one ODB prescription claim (51.5% of total cohort), with increasing percentages of people with at least one claim as marginalization level increased (40.7% in Q1 vs 65.8% in Q5). The number of any prescribed drugs that an individual was using also increased with increasing marginalization (mean(SD) 2.5(4.6) drugs in Q1 vs 5.6(6.8) drugs in Q5). Majority of people with SUD had at least one outpatient visit (84.8% of total cohort) with similar percentages across quintiles, however the number of outpatient visits increased with increasing quintile (mean(SD) 8.5(10.4) in Q1 vs 13.0(14.9) visits in Q5). More people had visits related to drugs compared to alcohol dependence, but as marginalization levels increased, the number of people with an alcohol-related visit decreased (8.5% in Q1 vs 5.8% in Q5) while the number with a drug-related visit increased (19.1% in Q1 vs 31.7% in Q5). The number of visits related to alcohol use was similar across the board (mean of 0.2 visits) while the number of drug use visits increased with increasing marginalization (mean(SD) 2.4 (7.7) in Q1 vs 6.0 (12.8) visits in Q5). 13.8% of Cohort 1 had at least one hospitalization (mean(SD) 0.2 (0.9) visits), with the highest proportion found in Q5 (16.9%) and lowest in Q2 (11.4%). Almost half had an ED visit, with Q5 having the highest proportion (49.8%) and Q2 the lowest (40.2%).

**Table 3:** Cohort 1 (pre-pandemic): Healthcare utilization outcomes stratified by overall marginalization quintile.

Variable	TOTAL	Q1	Q2	Q3	Q4	Q5
	N = 259,497^a^	N = 246	N = 39,070	N = 97,374	N = 98,957	N = 14,278
**At least one ODB claim, n(%)**						
Any drug	133,720 (51.5%)	100 (40.7%)	16,755 (42.9%)	46,477 (47.7%)	57,530 (58.1%)	9,392 (65.8%)
**N of drugs used based on ODB, mean(SD)**						
Any drug	3.7 (5.6)	2.5 (4.6)	2.6 (4.6)	3.2 (5.2)	4.4 (6.0)	5.6 (6.8)
**At least one mental health and addiction prescription claim, n(%)**						
Any drug	128,686 (49.6%)	104 (42.3%)	17,568 (45.0%)	46,627 (47.9%)	51,960 (52.5%)	7,765 (54.4%)
Benzodiazepine	49,421 (19.0%)	38 (15.4%)	6,966 (17.8%)	18,291 (18.8%)	20,065 (20.3%)	3,206 (22.5%)
Opioid agonist treatment	57,953 (22.3%)	29 (11.8%)	6,523 (16.7%)	19,868 (20.4%)	24,824 (25.1%)	3,512 (24.6%)
Opioid (non-OAT)	54,918 (21.2%)	43 (17.5%)	7,830 (20.0%)	20,096 (20.6%)	22,177 (22.4%)	3,584 (25.1%)
Stimulant	13,660 (5.3%)	15 (6.1%)	2,242 (5.7%)	5,119 (5.3%)	5,223 (5.3%)	742 (5.2%)
**At least one outpatient visit, n(%)**						
Any reason	219,937 (84.8%)	206 (83.7%)	33,347 (85.4%)	82,434 (84.7%)	84,287 (85.2%)	12,354 (86.5%)
Any mental health	91,602 (35.3%)	78 (31.7%)	13,987 (35.8%)	34,276 (35.2%)	35,693 (36.1%)	5,499 (38.5%)
Alcohol use	15,503 (6.0%)	21 (8.5%)	2,574 (6.6%)	6,157 (6.3%)	5,596 (5.7%)	824 (5.8%)
Drug use	73,475 (28.3%)	47 (19.1%)	8,610 (22.0%)	25,732 (26.4%)	30,918 (31.2%)	4,525 (31.7%)
**Number of outpatient visits, mean(SD)**						
Any reason	11.2 (13.8)	8.5 (10.4)	9.8 (12.6)	10.7 (13.3)	12.2 (14.6)	13.0 (14.9)
Any mental health	1.5 (4.2)	1.3 (3.2)	1.5 (3.9)	1.5 (4.3)	1.6 (4.2)	1.8 (4.5)
Alcohol use	0.2 (1.3)	0.2 (1.2)	0.2 (1.3)	0.2 (1.4)	0.2 (1.3)	0.2 (1.2)
Drug use	5.1 (11.6)	2.4 (7.7)	3.7 (9.9)	4.6 (11.0)	5.9 (12.5)	6.0 (12.8)
**At least one hospitalization, n(%)**						
Any reason	35,849 (13.8%)	34 (13.8%)	4,448 (11.4%)	12,700 (13.0%)	14,676 (14.8%)	2,417 (16.9%)
Any mental health	8,691 (3.3%)	7 (2.8%)	1,053 (2.7%)	3,078 (3.2%)	3,642 (3.7%)	600 (4.2%)
Any substance use	7,495 (2.9%)	9 (3.7%)	981 (2.5%)	2,763 (2.8%)	3,021 (3.1%)	473 (3.3%)
Alcohol use	3,676 (1.4%)	*1–5	523 (1.3%)	1,352 (1.4%)	1,441 (1.5%)	252 (1.8%)
Opioid use	459 (0.2%)	*1–5	60 (0.2%)	171 (0.2%)	181 (0.2%)	36 (0.3%)
Other drug use^b^	2,365 (0.9%)	*1–5	281 (0.7%)	877 (0.9%)	985 (1.0%)	134 (0.9%)
Other substance use^c^	9 (0.0%)	0 (0.0%)	*1–5	*1–5	*1–5	*1–5
**N of hospitalizations, mean(SD)**						
Any reason	0.2 (0.9)	0.2 (0.9)	0.2 (0.8)	0.2 (0.8)	0.3 (0.9)	0.3 (1.0)
Any mental health	0.1 (0.4)	0.0 (0.2)	0.0 (0.4)	0.0 (0.4)	0.1 (0.4)	0.1 (0.4)
Any substance use	0.0 (0.3)	0.0 (0.2)	0.0 (0.3)	0.0 (0.3)	0.0 (0.3)	0.0 (0.3)
**At least one ED visit, n(%)**						
Any reason	118,898 (45.8%)	107 (43.5%)	15,708 (40.2%)	43,303 (44.5%)	47,594 (48.1%)	7,104 (49.8%)
Any mental health	20,841 (8.0%)	14 (5.7%)	2,573 (6.6%)	7,433 (7.6%)	8,571 (8.7%)	1,379 (9.7%)
Any substance use	22,776 (8.8%)	18 (7.3%)	2,628 (6.7%)	7,940 (8.2%)	9,334 (9.4%)	1,676 (11.7%)
Alcohol use	13,413 (5.2%)	7 (2.8%)	1,598 (4.1%)	4,763 (4.9%)	5,285 (5.3%)	986 (6.9%)
Opioid use	3,383 (1.3%)	*1–5	364 (0.9%)	1,140 (1.2%)	1,418 (1.4%)	247 (1.7%)
Other drug use^b^	8,862 (3.4%)	10 (4.1%)	960 (2.5%)	3,023 (3.1%)	3,821 (3.9%)	694 (4.9%)
Other substance use^c^	34 (0.0%)	0 (0.0%)	*1–5	*17–21	12 (0.0%)	0 (0.0%)
**N of ED visits, mean(SD)**						
Any reason	1.5 (4.3)	1.1 (2.1)	1.1 (2.8)	1.4 (3.8)	1.6 (4.8)	1.9 (5.6)
Any mental health	0.2 (1.0)	0.1 (0.4)	0.1 (0.9)	0.1 (1.0)	0.2 (1.1)	0.2 (1.3)
Any substance use	0.2 (1.4)	0.1 (0.8)	0.1 (0.9)	0.2 (1.3)	0.2 (1.6)	0.3 (2.0)
**At least one urine drug screening test, n(%)**	64,661 (24.9%)	39 (15.9%)	7,652 (19.6%)	22,644 (23.3%)	27,929 (28.2%)	4,068 (28.5%)
**N of urine drug screening tests, mean(SD)**	9.0 (20.0)	4.6 (14.6)	6.3 (16.7)	8.2 (19.2)	10.4 (21.3)	10.2 (21.2)

^a^ Includes N = 9,572 with missing marginalization score (not reported)

^b^ “Other drug use” includes cannabis, sedative/hypnotics, cocaine, other stimulants, hallucinogens, volatile solvents

^c^ “Other substance use” includes nicotine, tobacco, other non-psychoactive substances such as antacids, vitamins etc.

*Small cells (n<5) are suppressed to prevent re-identification of individuals

Abbreviations: ODB = Ontario Drug Benefit, OAT = opioid agonist treatment, ED = emergency department

Healthcare utilization in Cohort 2 (during pandemic) is reported in Table 4. Q5 had the highest percentage of people with an ODB claim (61.2%) while Q2 had the lowest (33.3%). Similarly, people in Q5 were using the most drugs on average (mean(SD) 5.1(6.5)) while those in Q2 were using the fewest (mean(SD) 2.2(4.4)). Most people had at least one outpatient visit (82.4%) with similar percentages across the quintiles. However, the number of outpatient visits increased with increasing marginalization (mean(SD) 9.5(12.6) visits in Q1 vs 13.4(16.1) in Q5). The number of people with an alcohol use visit was similar across the board (5–6%), while the number with a drug use visit increased with increasing marginalization (18.7% in Q1 vs 32.4% in Q5). Similarly, average number of alcohol use visits remained at 0.2 across quintiles, while drug use visits increased with increasing marginalization (mean(SD) 3.0(8.8) in Q1 vs 6.3(13.5) in Q5). 13.5% of the cohort had a hospitalization (mean(SD) 0.2(0.9) visits), with the proportion increasing as marginalization levels increased (9.0% in Q1 vs 17.2% in Q5). Almost half of the cohort had at least one ED visit (mean(SD) 1.4(4.0) visits), with the highest proportion in Q5 (49.3%) and lowest in Q2 (38.8%).

**Table 4:** Cohort 2 (during pandemic): Healthcare utilization outcomes stratified by overall marginalization quintile.

Variable	TOTAL	Q1	Q2	Q3	Q4	Q5
	N = 276,459^a^	N = 278	N = 42,543	N = 104,703	N = 103,137	N = 14,729
**At least one Ontario Drug Benefit claim, n(%)**						
Any drug	123,656 (44.7%)	121 (43.5%)	14,168 (33.3%)	43,201 (41.3%)	53,938 (52.3%)	9,017 (61.2%)
**N of drugs used based on ODB, mean(SD)**						
Any drug	3.2 (5.3)	2.9 (5.4)	2.2 (4.4)	2.9 (5.0)	3.9 (5.7)	5.1 (6.5)
**At least one mental health and addiction prescription claim, n(%)**						
Any drug	130,514 (47.2%)	120 (43.2%)	18,381 (43.2%)	47,999 (45.8%)	50,982 (49.4%)	7,609 (51.7%)
Benzodiazepine	47,642 (17.2%)	44 (15.8%)	6,958 (16.4%)	18,090 (17.3%)	18,759 (18.2%)	2,925 (19.9%)
Opioid agonist treatment	61,758 (22.3%)	45 (16.2%)	7,155 (16.8%)	21,403 (20.4%)	25,341 (24.6%)	3,773 (25.6%)
Opioid (non-OAT)	52,784 (19.1%)	52 (18.7%)	7,416 (17.4%)	19,552 (18.7%)	21,122 (20.5%)	3,329 (22.6%)
Stimulant	17,280 (6.3%)	20 (7.2%)	3,026 (7.1%)	6,730 (6.4%)	6,241 (6.1%)	853 (5.8%)
**At least one outpatient visit, n(%)**						
Any reason	227,846 (82.4%)	233 (83.8%)	35,426 (83.3%)	86,451 (82.6%)	85,416 (82.8%)	12,362 (83.9%)
Any mental health	96,244 (34.8%)	96 (34.5%)	15,350 (36.1%)	36,708 (35.1%)	36,342 (35.2%)	5,467 (37.1%)
Alcohol use	16,517 (6.0%)	16 (5.8%)	2,779 (6.5%)	6,684 (6.4%)	5,830 (5.7%)	818 (5.6%)
Drug use	77,170 (27.9%)	52 (18.7%)	9,435 (22.2%)	27,395 (26.2%)	31,435 (30.5%)	4,766 (32.4%)
**Number of outpatient visits, mean(SD)**						
Any reason	11.4 (14.6)	9.5 (12.6)	10.2 (13.4)	11.0 (14.2)	12.2 (15.3)	13.4 (16.1)
Any mental health	1.6 (4.6)	1.2 (2.5)	1.6 (4.3)	1.6 (4.7)	1.6 (4.5)	1.9 (5.5)
Alcohol use	0.2 (1.6)	0.2 (1.2)	0.2 (2.0)	0.2 (1.7)	0.2 (1.4)	0.2 (1.4)
Drug use	5.0 (12.0)	3.0 (8.8)	3.7 (10.5)	4.6 (11.5)	5.8 (12.9)	6.3 (13.5)
**At least one hospitalization, n(%)**						
Any reason	37,244 (13.5%)	25 (9.0%)	4,602 (10.8%)	13,175 (12.6%)	15,109 (14.6%)	2,536 (17.2%)
Any mental health	8,863 (3.2%)	*1–5	1,113 (2.6%)	3,123 (3.0%)	3,612 (3.5%)	670 (4.5%)
Any substance use	8,223 (3.0%)	*1–5	1,015 (2.4%)	2,992 (2.9%)	3,316 (3.2%)	536 (3.6%)
Alcohol use	3,688 (1.3%)	*1–5	472 (1.1%)	1,349 (1.3%)	1,481 (1.4%)	235 (1.6%)
Opioid use	965 (0.3%)	0 (0.0%)	102 (0.2%)	369 (0.4%)	388 (0.4%)	67 (0.5%)
Other drug use^b^	2,425 (0.9%)	*1–5	284 (0.7%)	839 (0.8%)	1,013 (1.0%)	164 (1.1%)
Other substance use^c^	26 (0.0%)	0 (0.0%)	0 (0.0%)	9 (0.0%)	*12–16	*1–5
**N of hospitalizations, mean(SD)**						
Any reason	0.2 (0.9)	0.2 (0.7)	0.2 (0.7)	0.2 (0.8)	0.3 (1.0)	0.3 (1.0)
Any mental health	0.0 (0.4)	0.0 (0.2)	0.0 (0.3)	0.0 (0.4)	0.1 (0.4)	0.1 (0.4)
Any substance use	0.0 (0.3)	0.0 (0.1)	0.0 (0.3)	0.0 (0.3)	0.0 (0.3)	0.1 (0.4)
**At least one ED visit, n(%)**						
Any reason	122,198 (44.2%)	115 (41.4%)	16,520 (38.8%)	44,909 (42.9%)	47,800 (46.3%)	7,255 (49.3%)
Any mental health	20,825 (7.5%)	8 (2.9%)	2,541 (6.0%)	7,449 (7.1%)	8,460 (8.2%)	1,394 (9.5%)
Any substance use	23,385 (8.5%)	14 (5.0%)	2,599 (6.1%)	8,198 (7.8%)	9,426 (9.1%)	1,658 (11.3%)
Alcohol use	12,145 (4.4%)	9 (3.2%)	1,429 (3.4%)	4,364 (4.2%)	4,657 (4.5%)	763 (5.2%)
Opioid use	4,747 (1.7%)	*1–5	449 (1.1%)	1,587 (1.5%)	2,058 (2.0%)	358 (2.4%)
Other drug use^b^	9,549 (3.5%)	*1–5	960 (2.3%)	3,264 (3.1%)	4,043 (3.9%)	808 (5.5%)
Other substance use^c^	28 (0.0%)	0 (0.0%)	0 (0.0%)	7 (0.0%)	15 (0.0%)	*1–5
**N of ED visits, mean(SD)**						
Any reason	1.4 (4.0)	1.1 (3.1)	1.0 (2.5)	1.3 (3.8)	1.6 (4.5)	1.8 (4.7)
Any mental health	0.2 (1.1)	0.0 (0.2)	0.1 (0.6)	0.1 (1.0)	0.2 (1.2)	0.2 (1.4)
Any substance use	0.2 (1.3)	0.1 (0.3)	0.1 (0.7)	0.2 (1.3)	0.2 (1.3)	0.3 (1.8)
**At least one urine drug screening test, n(%)**	61,241 (22.2%)	47 (16.9%)	7,323 (17.2%)	21,646 (20.7%)	25,821 (25.0%)	3,915 (26.6%)
**N of urine drug screening tests, mean(SD)**	7.0 (16.9)	4.8 (14.4)	5.0 (14.1)	6.4 (16.2)	8.1 (18.1)	8.2 (17.9)

^a^ Includes N = 11,069 with missing marginalization score (not reported)

^b^ “Other drug use” includes cannabis, sedative/hypnotics, cocaine, other stimulants, hallucinogens, volatile solvents

^c^ “Other substance use” includes nicotine, tobacco, other non-psychoactive substances such as antacids, vitamins etc.

*Small cells (n<5) are suppressed to prevent re-identification of individuals

Abbreviations: ODB = Ontario Drug Benefit, OAT = opioid agonist treatment, ED = emergency department

## Discussion

This population-based, cross-sectional study compared baseline characteristics and healthcare utilization across marginalization levels among Ontarians with SUD before and during the COVID-19 pandemic. Over 40% of people with SUD belonged to the two highest marginalization quintiles. Those most marginalized were more likely to be older, male, urban residents, and have comorbidities. The use of services such as outpatient visits, prescription claims, and ED visits was high among people with SUD, with increasing marginalization associated with increasing use. Comparisons before and during the pandemic revealed similarities in baseline characteristics and most healthcare service use between the two cohorts.

The strong association between marginalization and SUD has been well documented in the literature. For example, those with lower income are more likely to encounter substance use issues compared to higher income individuals [18]. Marginalized individuals, such as those who are experiencing homelessness or are vulnerably housed, often suffer from psychological trauma and isolation, and may turn to substance use as a coping mechanism [19]. Individuals from marginalized communities may also use substances as a way of assimilating with their peers [19]. Ethnic marginalization can increase isolation, mental distress and subsequent substance use [20]. Further, those experiencing intersectional discrimination, such as ethnic and gender discrimination, may be exposed to increased risk of SUD [21]. The finding that urban residence increased with greater marginalization among people with SUD aligns with overall census data citing higher poverty levels among large urban areas due to higher costs of living [22]. Such circumstances may, in turn, also facilitate easier access to substances. People from marginalized communities have been reported to have a higher burden of chronic diseases [23], and we would expect these trends to remain even among the SUD population, as we have found. We found high levels of healthcare use for most but not all services in the SUD population. Although hospitalizations were rarer, most of the population had at least one outpatient interaction both pre and during pandemic, regardless of marginalization level. We also found that marginalized individuals documented a higher frequency of outpatient visits compared to the less marginalized, which speaks to greater health needs among structurally marginalized people with SUD [24]. These findings also suggest that people are still able to access care despite the barriers imposed by their marginalization status as well as COVID-19 [25], with the latter possibly due to the shift towards telemedicine [26]. However, despite the apparent access to primary care, almost half visited the ED, with higher marginalization associated with higher percentage of individuals having at least one ED visit both before and during the pandemic. The reason for this is unclear, but may be related to wait times for seeing a primary care provider.

Around half of the SUD population had at least one ODB claim, indicating that a significant proportion of people with SUD are covered by the Ontario Drug Benefit program, which is only available to those from select demographic groups, such as those ≤ 24 years and ≥ 65 years, people receiving specialized care, who have disabilities, or have demonstrated financial need [27]. Given that most of our cohort is between age 25–64 (see S4 Table), this suggests that much of the SUD population have more severe health or social/economic needs that allow them to qualify for ODB. This aligns with our finding that the percentage of people with SUD with ODB claims as well as the average number of claims increases with increasing marginalization level. Interestingly, the percentage of people with prescription claims declined during the pandemic (51.5% vs 44.7%), despite other healthcare utilization remaining relatively unchanged. Although the reason for this is unclear, it is possible that this difference is attributed to drug shortages that were caused by the pandemic [28] or changes in physician prescribing patterns during this time. We also noted a slight decrease in the percentage of people with an outpatient visit during the pandemic compared to before (82.4% vs 84.8%), which perhaps indicates that individuals may not have been refilling their prescriptions.

The percentage of people with a hospitalization or ED visit due to alcohol use was similar to that for drug use, however the percentage with an outpatient visit due to alcohol use was significantly lower than drug use. Because alcohol is a legal substance, widely accessible, and deemed more socially acceptable than drugs, it is possible that individuals with alcohol dependence view alcohol as less harmful and therefore are more likely to delay seeking care until they require urgent and severe medical attention compared to those with drug dependence. Furthermore, we found that there was no consistent pattern in the percentage of people who had an alcohol-related outpatient visit or the average number of alcohol-related visits across quintiles. However, the percentage with a drug-related outpatient visit and the average number of drug-related visits increased with increasing marginalization level, which was a consistent finding in both cohorts. This suggests that alcohol use may not be as strongly associated with marginalization as drug use [29], as prior research found that individuals with higher socioeconomic status (SES) may have similar or even more alcohol use than those with low SES [30], whereas risk for drug use is often greater among marginalized populations [31]. Nevertheless, marginalized individuals are more likely to suffer greater severity of alcohol-related consequences [30], as evident in the increasing proportion of alcohol-related ED visits and hospitalizations with increasing marginalization in our study. Alcohol-related health service use appeared to be similar before and during the pandemic, possibly because our pandemic cohort began in June 2021. The spike in alcohol consumption likely occurred in the first year of COVID, and subsequently returned to pre-pandemic levels as the pandemic progressed [32].

Limitations of this study include the lack of granularity that accompanies the use of administrative datasets, which prevents us from assessing clinical details such as the content or appropriateness of the services used. There were no available databases that systematically captured community-based SUD programs, therefore these services were not included in our study. Due to the variability of substance use in the population, our inclusion criteria may have inadvertently captured those who only experienced the harms of substance use and have not necessarily received a diagnosis of SUD. However, our algorithm for defining SUD focused on those who presented with more serious instances of substance use, hence most of our population should include those who meet the criteria for SUD. Furthermore, administrative records only capture those with active disease or those seeking help from the healthcare system, and as such, our findings may be an underestimate of the true prevalence of SUD in the population. Second, our study leverages marginalization quintiles which are based on area-level approximations and require probabilistic matching of postal codes to dissemination areas. This is generally less accurate in rural settings where postal codes cover larger areas. Furthermore, some dissemination areas are not assigned an Ontario Marginalization Index score due to incomplete census enumeration in that area (such as rural and Indigenous populations), and therefore these residents are not well captured in the index [33]. Lastly, our study compares two cross-sections in time, which do not capture incident cases of SUD. Given the dynamic nature of SUD, future studies can consider incorporating longitudinal designs to document changes in health service usage across levels of marginalization in the SUD population.

## Conclusions

This study compares baseline clinical and sociodemographic characteristics as well as healthcare utilization among people with SUD across levels of marginalization before and during the pandemic. Increasing marginalization generally appeared to be associated with increased healthcare service use and greater health needs. Baseline characteristics and most healthcare use remained relatively similar in this population before and during COVID. Findings from this study help to further our understanding of the intricate relationship between marginalization, SUD, and health outcomes, which can aid policy makers in guiding the development and implementation of interventions for this vulnerable population, particularly in the dynamic post-COVID landscape.

## Supporting information

S1 Table ICES databases. (DOCX)

S2 Table Definition of substance use disorder. (DOCX)

S3 Table Percentage of people with SUD by region. (DOCX)

S4 Table Age distribution of Cohort 1 and 2 by marginalization quintile. (DOCX)

## Abbreviations

DAD: Discharge Abstract Database
ED: emergency department
ICES: Institute for Clinical Evaluative Sciences
NACRS: National Ambulatory Care Reporting System
NMS: Narcotics Monitoring System
OAT: opioid agonist treatment
ODB: Ontario Drug Benefit
OHIP: Ontario Health Insurance Plan
OMHRS: Ontario Mental Health Reporting System
ONMARG: Ontario Marginalization Index
PCCF: Postal Code Conversion File
PHIPA: Personal Health Information Protection Act
REB: Research Ethics Board
RPDB: Registered Persons Database
SD: standard deviation
SDS: Same Day Surgery
SES: socioeconomic status
SUD: substance use disorder
