# Supplementary material for: Impact of marginalization on characteristics and healthcare utilization among people with substance use disorder in Ontario, Canada, before and during the COVID-19 pandemic: A cross-sectional study
Source: PLoS One. 2024 Oct 25;19(10):e0312270. doi: 10.1371/journal.pone.0312270 (PMC11508079; doi:10.1371/journal.pone.0312270)
Supplement: S1 Table — (DOCX) [file pone.0312270.s001.docx]

**S1 Table. ICES Databases**

| **Database** | **Description of data** |
| --- | --- |
| CIHI-Discharge Abstract Database (DAD) | Captures administrative, clinical and demographic information on hospital discharges |
| CIHI-Same Day Surgery (SDS) | Contains records of same day surgeries, such as procedures undergone as well as clinical information about the individual |
| CIHI-National Ambulatory Care Reporting System (NACRS) | Contains data for hospital-based and community-based ambulatory care, such as day surgery, outpatient and community-based clinics, and emergency departments |
| Ontario Health Insurance Plan (OHIP) | Contains data on all physician claims provided to patients covered under the Ontario Health Insurance Plan |
| Ontario Mental Health Reporting System (OMHRS) | Contains data on all individuals receiving adult mental health services in Ontario |
| Ontario Drug Benefit (ODB) | Contains Ontario Drug Benefit (ODB) information, including recipients, payment, claims, and pharmacy and practitioner information |
| Registered Persons Database (RPDB) | Contains information on persons registered under the Ontario Health Insurance Plan (OHIP) and who are eligible for the Ontario Drug Program |
| Postal Code Conversion File (PCCF) | Provides a link between the six-character postal codes and census geographic areas such as dissemination areas, census tracts and others |
| Narcotics Monitoring System (NMS) | Captures information from dispensers (e.g. pharmacies, dispensing physicians) about all prescribed monitored drugs dispensed to people in Ontario |
| Ontario Marginalization Index (ONMARG) | Contains data on ON-Marg, which measures multiple axes of deprivation in Ontario, including economic, ethno-racial, age-based and social marginalization |
| ICES validated disease registries: ASTHMA, CHF, COPD, DEMENTIA, HIV, HYPER, ODD | Contains clinical information on individuals diagnosed with a chronic disease |
