# Supplementary material for: Impact of marginalization on characteristics and healthcare utilization among people with substance use disorder in Ontario, Canada, before and during the COVID-19 pandemic: A cross-sectional study
Source: PLoS One. 2024 Oct 25;19(10):e0312270. doi: 10.1371/journal.pone.0312270 (PMC11508079; doi:10.1371/journal.pone.0312270)
Supplement: S2 Table — (DOCX) [file pone.0312270.s002.docx]

**S2 Table. Definition of Substance Use Disorder**

| **Criteria** | **Codes used to define substance use** |
| --- | --- |
| Emergency department visit or inpatient hospitalization with substance use disorder diagnosis | **Any** claim with the following diagnosis codes in the 5-years before (including) the index date:   - ICD-9 codes [OMHRS]: 291.x, 292.x, 303.x, 304.x - ICD-10 codes [NACRS, DAD, OMHRS]: F10-19, F55 |
| Outpatient visit with substance use disorder diagnosis | **≥3 claims** within the 5-years before (including) the index date to fulfil this criterion (ONLY keep one claim per person per day):   - ICD-9 codes/DXCODES [OHIP]: 291.x, 292.x, 303.x, 304.x |
| Opioid agonist treatment use | **Any** prescription claim for opioid agonist treatment (OAT) in NMS and/or ODB within the 5-years before (including) the index date:   - Methadone - Buprenorphine |
