# Supplementary material for: Impact of marginalization on characteristics and healthcare utilization among people with substance use disorder in Ontario, Canada, before and during the COVID-19 pandemic: A cross-sectional study
Source: PLoS One. 2024 Oct 25;19(10):e0312270. doi: 10.1371/journal.pone.0312270 (PMC11508079; doi:10.1371/journal.pone.0312270)
Supplement: S3 Table — (DOCX) [file pone.0312270.s003.docx]

**S3 Table. Percentage of people with SUD by region**

| **Cohort 1 (before pandemic)** | | | |
| --- | --- | --- | --- |
| **Region of residence** | **Number of people with SUD in June 2018 – June 2019** | **Population count as of July 2019** | **% of people with SUD** |
| Central | 59,626 | 4,767,090 | 1.3% |
| East | 59,358 | 3,587,358 | 1.7% |
| North | 29,973 | 810,415 | 3.7% |
| Toronto | 29,070 | 1,336,763 | 2.2% |
| West | 81,470 | 4,043,075 | 2.0% |
| **Cohort 2 (during pandemic)** | | | |
| **Region of residence** | **Number of people with SUD in June 2021 – June 2022** | **Population count as of July 2022** | **% of people with SUD** |
| Central | 62,434 | 5,113,839 | 1.2% |
| East | 63,496 | 3,742,517 | 1.7% |
| North | 33,392 | 797,428 | 4.2% |
| Toronto | 29,275 | 1,440,644 | 2.0% |
| West | 87,862 | 4,133,902 | 2.1% |
