# Supplementary material for: Impact of marginalization on characteristics and healthcare utilization among people with substance use disorder in Ontario, Canada, before and during the COVID-19 pandemic: A cross-sectional study
Source: PLoS One. 2024 Oct 25;19(10):e0312270. doi: 10.1371/journal.pone.0312270 (PMC11508079; doi:10.1371/journal.pone.0312270)
Supplement: S4 Table — (DOCX) [file pone.0312270.s004.docx]

**S4 Table. Age distribution of Cohort 1 and 2 by marginalization quintile**

| **COHORT 1 (before pandemic)** | | | | | | |
| --- | --- | --- | --- | --- | --- | --- |
| **Age group** | **Total** | **Q1** | **Q2** | **Q3** | **Q4** | **Q5** |
| ≤24 years | 47,482 (18.3%) | 60 (24.4%) | 10,016 (25.6%) | 18,488 (19.0%) | 15,161 (15.3%) | 1,715 (12.0%) |
| 25-44 years | 115,736 (44.6%) | 93 (37.8%) | 15,847 (40.6%) | 43,590 (44.8%) | 45,008 (45.5%) | 5,943 (41.6%) |
| 45-64 years | 76,462 (29.5%) | 78 (31.7%) | 10,408 (26.6%) | 27,705 (28.5%) | 31,117 (31.4%) | 5,159 (36.1%) |
| 65+ years | 19,817 (7.6%) | 15 (6.1%) | 2,799 (7.2%) | 7,591 (7.8%) | 7,671 (7.8%) | 1,461 (10.2%) |
| **COHORT 2 (during pandemic)** | | | | | | |
| **Age group** | **Total** | **Q1** | **Q2** | **Q3** | **Q4** | **Q5** |
| ≤24 years | 41,937 (15.2%) | 61 (21.9%) | 9,085 (21.4%) | 16,331 (15.6%) | 13,059 (12.7%) | 1,540 (10.5%) |
| 25-44 years | 130,128 (47.1%) | 92 (33.1%) | 18,403 (43.3%) | 49,312 (47.1%) | 49,335 (47.8%) | 6,480 (44.0%) |
| 45-64 years | 81,173 (29.4%) | 95 (34.2%) | 11,586 (27.2%) | 30,024 (28.7%) | 32,038 (31.1%) | 5,091 (34.6%) |
| 65+ years | 23,221 (8.4%) | 30 (10.8%) | 3,469 (8.2%) | 9,036 (8.6%) | 8,705 (8.4%) | 1,618 (11.0%) |
